# Cumulative psychosocial factors are associated with cardiovascular disease risk factors and management among African Americans in the Jackson Heart Study

**DOI:** 10.1186/s12889-020-08573-0

**Published:** 2020-04-28

**Authors:** Mario Sims, Lá Shauntá M. Glover, Samson Y. Gebreab, Tanya M. Spruill

**Affiliations:** 1 Department of Medicine, University of Mississippi Medical Center, 350 W. Woodrow Wilson Avenue, Jackson, MS 39213, USA; 2 Department of Epidemiology, University of North Carolina at Chapel Hill, Chapel Hill, NC USA; 3 National Institutes of Health, Rockville, MD USA; 4 Department of Population Health, NYU School of Medicine, New York, USA

**Keywords:** Psychosocial factors, Obesity, Hypertension, Diabetes, African Americans, Jackson Heart Study

## Abstract

**Background:**

Racial disparities in cardiovascular disease (CVD) have been attributed in part to negative psychosocial factors. Prior studies have demonstrated associations between individual psychosocial factors and CVD risk factors, but little is known about their cumulative effects.

**Methods:**

Using the Jackson Heart Study, we examined the cross-sectional associations of cumulative psychosocial factors with CVD risk factors among 5306 African Americans. We utilized multivariable Poisson regression to estimate sex-stratified prevalence ratios (PR 95% confidence interval-CI) of obesity, hypertension and diabetes prevalence and hypertension and diabetes control with negative affect (cynicism, anger-in, anger-out, depressive symptoms and cumulative negative affect) and stress (global stress, weekly stress, major life events-MLEs and cumulative stress), adjusting for demographics, socioeconomic status, and behaviors.

**Results:**

After full adjustment, high (vs. low) cumulative negative affect was associated with prevalent obesity among men (PR 1.36 95% CI 1.16–1.60), while high (vs. low) cumulative stress was similarly associated with obesity among men and women (PR 1.24 95% CI 1.01–1.52 and PR 1.13 95% CI 1.03–1.23, respectively). Psychosocial factors were more strongly associated with prevalent hypertension and diabetes among men than women. For example, men who reported high cynicism had a 12% increased prevalence of hypertension (PR 1.12, 95% CI 1.03–1.23). Psychosocial factors were more strongly associated with lower hypertension and diabetes control for women than men. Women who reported high (vs. low) cynicism had a 38% lower prevalence of hypertension control (PR 0.62, 95% CI 0.46–0.84).

**Conclusions:**

Cumulative psychosocial factors were associated with CVD risk factors and disease management among African Americans. The joint accumulation of psychosocial factors was more associated with risk factors for men than women.

## Background

Research has shown that African Americans suffer a disproportionate burden of cardiovascular diseases (CVD) than other groups [1]. Findings from the Jackson Heart Study (JHS) reported that diabetes prevalence was 20% for women and 16% for men, while diabetes control among diabetics was greater for women [2]. While hypertension prevalence in the JHS was 65% for women and 60% for men, hypertension control was greater for women than men [3]. Obesity in the JHS was over 60% for women and 40% for men [4]. CVD disparities may be influenced by non-clinical risk factors, such as psychosocial factors.

Psychosocial risk factors are associated with CVD development and progression in the broader population. In meta-analyses, anger and hostility [5] and depression [6] were associated with coronary heart disease (CHD) in initially healthy samples. Depression has also been associated with a greater risk of becoming obese in meta-analytic studies [7]. Additionally, psychosocial stress is associated with a greater risk of hypertension [8] and a greater risk of poor diabetes control [9].

Although meta-analytic studies have investigated the association of psychosocial risk factors with CVD and related morbidities in the broader population, there are greater disparities in psychosocial stressors among minorities, specifically, African Americans [10], which may contribute to disparities in CVD [11, 12]. One study found that high anger expression, depressive symptoms, and chronic stress were associated with progressing from optimal to stage 1 or 2 hypertension among African Americans over a 5-year period [13]. Another study found that African Americans who reported high cynicism, anger, depressive symptoms and stress smoked more and reported fewer hours of sleep [14].

Associations of multiple psychosocial factors and their cumulative effects with CVD risk factors have been understudied in large samples of African Americans. It is likely that these factors act together to affect disease prevalence and management. The associations with hypertension and diabetes control have implications for managing chronic diseases through considering non-traditional risk factors. Utilizing the JHS, we hypothesize that high cumulative negative affect (cynicism, anger, depressive symptoms) and cumulative stressors (global, weekly and major life events) are positively associated with obesity, hypertension and diabetes and negatively associated with hypertension and diabetes control and that associations vary by sex.

## Methods

The JHS is a community-based study of CVD among non-institutionalized African Americans living in the tri-county (Hinds, Rankin and Madison) area of Jackson, MS. The target sample size for the cohort study was 5500 participants among eligible African Americans in the Jackson, MS metropolitan area. Participants were sampled from four recruitment pools at baseline (2000–2004): (1) Jackson participants of the Atherosclerosis Risk in Communities (ARIC) study (30%); (2) participants randomly sampled from the Mississippi Department of Transportation Driver’s License and Identification List (17%); (3) volunteers that signed up for the study (22%); (4) family members of participants who agreed to be a part of the study (31%). The total sample included 5306 participants 35–84 years old (3371 women and 1935 men) [15]. Further details about recruitment, data collection and study variables are described elsewhere [16]. The study was approved by the institutional review boards of the University of Mississippi Medical Center, Jackson State University, and Tougaloo College, and participants provided written informed consent.

Outcome measures include obesity, hypertension prevalence and control, and diabetes prevalence and control. Body mass index (BMI) was derived from in-clinic weight and height measurements (kg/m [2]). *Obesity* was defined as BMI > 30. Trained staff conducted 2 blood pressure (BP) measurements in the right arm, and 1 min elapsed between the 2 measurements using a random zero sphygmomanometer (Hawksley and Sons Ltd., Lancing, UK) at exam 1. These 2 BP measurements were averaged for analysis. *Hypertension* was defined as systolic blood pressure ≥ 140 mmHg, diastolic blood pressure ≥ 90 mmHg, or being on antihypertensive medications, or physician diagnosis of hypertension. *Hypertension control* was defined as BP < 140/90 mmHg among those treated for hypertension. *Type 2 diabetes* was defined as fasting glucose ≥126 mg/dL, or HbA1c ≥ 6.5%, or use of diabetic medication, or physician diagnosis. *Diabetes control* was defined as HbA1c levels < 7.0% among diabetics.

Psychosocial measures included cynical distrust, anger-in and -out, depressive symptoms, global stress, weekly stress inventory (WSI), and major life events (MLEs). Items 1–13 of the Cook-Medley Hostility Scale [17] measure *cynicism* where participants were asked to answer “true or false” on such items “… It is safer to trust nobody.” We calculated a total score (range 0–13), where higher scores indicate higher distrust (α = 0.76). *Anger* was assessed using a validated scale that measured anger-in and -out (both 8 items) [18]. Participants were asked how often they reacted to such items as “I express my anger” that were rated from almost never (1) to almost always (4) Anger-in and -out scores ranged from 0 to 23 (anger-in; α = 0.77) and 0 to 22 (anger-out; α = 0.77), where higher scores indicate higher anger. *Depressive symptoms* were measured using the 20-item Centers for Epidemiologic Studies Depression (CES-D) scale, where participants were asked about their mood, responding to items (“I was bothered by things that … don’t bother me”) about how often they felt this way. Items rated from 0 (“rarely/none of the time”) to 3 (“most/all of the time”). This scale ranged from 0 to 60 with higher scores reflecting greater depressive symptoms (α = 0.82).

The global *stress* scale was created for the JHS and adopted from the Perceived Stress Scale. Participants rated the extent of stress perceived in 8 domains (e.g., employment, meeting basic needs) over a twelve-month period. Choices ranged from “not stressful” to “very stressful” and total summed scores ranged from 0 to 24, where higher scores denote higher stress (α = 0.72). The *WSI*, developed by Jones and Brantley [19], is an 87-item questionnaire that measured the occurrence of minor irritants (e.g., work tasks, financial challenge) during the past week, and the extent to which they caused stress (1 = not stressful; 7 = extremely stressful). Two components of the WSI scale were measured: 1) WSI-events, or number of stressful events (range 0–87), where higher scores indicate higher stress; and 2) WSI-impact, or sum of subjective ratings assigned to events (range 0–493; α = 0.98). Using an 11-item life events inventory, *MLEs* measured whether respondents experienced major events in the last 12 months by answering “yes” or “no” to a series of items (e.g., serious illness, victim of assault). The total score was calculated by summing the “yes” responses (range 1–11). Because MLE is not a true scale, Cronbach’s alpha was not calculated.

Each psychosocial measure was classified in tertiles (range 1–3) to examine possible non-linear associations. Additionally, we investigated negative affect and stress measures jointly by constructing cumulative indices for each dimension [13, 14]. *Cumulative negative affect* was constructed by summing the tertiles of cynical distrust, anger-in and -out and depressive symptoms to get a total score (range 4–12). *Cumulative stress* was constructed by summing the tertiles of global stress, WSI-events, WSI-impact and MLEs to get a total score (range 4–12).

Covariates included age (continuous), sex [men/women (referent)], education, income, health behaviors and BMI. Education was classified as less than high school (referent), high school graduate to some college, or college graduate and above. Household income was composed of the following categories: poor (referent), lower-middle, upper-middle, and affluent categories that correspond with < poverty level, 1–1.6 times the poverty level, > 1.6 but < 3 times the poverty level, and 3.5+ times the poverty level, respectively. Health behaviors included physical activity, smoking, diet, and alcohol consumption. Participants had “ideal physical activity” if they reported more than 150 min/week of moderate physical activity or more than 75 min of vigorous physical activity; participants had “non-ideal physical activity” (referent) if they reported less than 150 min of moderate physical activity and less than 75 min of vigorous physical activity. Participants who reported they never smoked or quit smoking more than a year prior to examination were categorized as “ideal”; those who were current smokers or had quit smoking less than 12 months prior to examination were considered “non-ideal” (referent). Based on the 158-item food frequency questionnaire (Delta Nutrition Intervention Research Initiative), diet was measured by percent calories from fat. Alcohol consumption was measured by participants responding “yes” or “no” whether they have consumed alcohol in the past 12 months.

### Statistical analysis

Descriptive statistics examined the psychosocial and CVD risk factors by sex, and differences were tested using chi-square or analysis of variance (ANOVA) tests for categorical and continuous variables, respectively. Multivariable prevalence regression estimated prevalence ratios (PR, 95% confidence interval-CI) of psychosocial factors with outcomes using robust standard errors. Because the prevalence of obesity, hypertension, diabetes, hypertension control and diabetes control was high in this sample, Poisson regression was used to estimate PRs of each outcome by psychosocial factors before and after adjustment for covariates [20]. Model 1 adjusted for age; Model 2 adjusted for model 1 plus education and income; Model 3 adjusted for model 2 plus smoking, physical activity, consumption of calories from fat, alcohol consumption, and BMI (except in the obesity analysis). Due to the large number of analyses, we only present the full models in the results. We present fully-adjusted associations of cumulative negative affect and cumulative stress with CVD risk factors in figures. As separate dimensions the negative affect measures were highly correlated (range 0.13–0.40, *p* < 0.0001) and the stress measures were highly correlated (range 0.13–0.90, *p* < 0.001); thus, all psychosocial factors were estimated in separate models to avoid multicollinearity. *P* values for trend, estimated for tertiles of each psychosocial factor, represented the linear trend in the association with each outcome across medium and high (vs. low) categories. As a caveat, there were statistically significant *p* values for trend (< 0.05) in the results below (where the PRs were not significant), which indicated a significant (positive or negative) dose-response effect of psychosocial factors on the outcomes regardless of whether the PRs were significant. A test for effect modification by sex demonstrated associations varied by sex (*p* values for interaction < 0.05); therefore, our analysis was stratified by sex [21].

**Table 1:** Distribution of psychosocial measures and CVD Risk factors among men and women, JHS, 2000–2004 (*n* = 4806)

Variable	Total	Men	Women	P Value
*Psychosocial Measures (Mean, SD)*				
Cynical Distrust	6.8 (3.2)	7.2 (3.1)	6.6 (6.5)	< 0.001
Anger in	5.5 (3.5)	5.6 (3.5)	5.4 (3.5)	0.05
Anger out	4.5 (3.1)	4.5 (3.1)	4.6 (3.2)	0.55
Depressive symptoms	10.9 (8.1)	9.7 (7.3)	11.5 (8.4)	< 0.001
Global Stress	5.1 (4.4)	4.5 (4.2)	5.5 (4.4)	< 0.001
WSI-Event	32.3 (22.8)	33.1 (24.2)	31.8 (22.1)	0.11
WSI-Impact	81.4 (81.6)	76.9 (80.0)	84.0 (82.4)	0.02
Major Life Events	3.5 (1.4)	3.3 (1.3)	3.6 (1.4)	< 0.001
Cumulative Negative Affect	7.64 (2.16)	7.63 (2.2)	7.64 (2.1)	0.91
Cumulative Stress	7.57 (2.25)	7.31 (2.2)	7.72 (2.3)	< 0.001
*CVD Risk factors (%)*				
Obesity	53.4	41.5	60.2	< 0.001
Hypertension	62.9	60.1	64.5	0.002
Diabetes	18.8	17.0	19.8	0.02
Hypertension Control^*^	26.6	23.3	28.7	0.002
Diabetes Control^**^	38.6	36.1	39.8	0.34

*P* values based on ANOVA tests and Chi Square Tests. Abbreviations: *WSI* Weekly Stress Inventory; *SD* Standard deviation; *BMI* Body mass index. Cumulative negative affect score (range 4–12) is the sum of the individual tertile scores (range 1–3) for cynical distrust, anger in, anger out, and depressive symptoms. Cumulative stress score (range 4–12) is the sum of the individual tertile scores (range 1–3) for global perceived stress, WSI-event, WSI-impact, and major life events

^*^The denominator for hypertension control includes all hypertensives on anti-hypertensive medications (*n* = 2654)

^**^The denominator for diabetes control includes all diabetics on anti-diabetic medications (*n* = 751)

**Table 2:** Prevalence Ratios (PR 95% CI) of Obesity, Hypertension and Diabetes by psychosocial measures among men and women, JHS (2000–2004)

*Psychosocial Measures*	Obesity (PR, 95% CI)		Hypertension (PR, 95% CI)		Diabetes (PR, 95% CI)	
	Men	Women	Men	Women	Men	Women
Cynical distrust						
Low (referent)	1.0	1.0	1.0	1.0	1.0	1.0
Medium	1.11 (0.97–1.27)	1.09 (1.03–1.16)*^a^	0.98 (0.90–1.07)	0.98 (0.94–1.03)	2.26 (1.56–3.29)*^a^	0.93 (0.81–1.07)
High	1.28 (1.12–1.47)*^a^	1.09 (1.02–1.17)*^a^	1.12 (1.03–1.23)*^a^	0.93 (0.87–0.98)^Ɨ^	3.28 (2.26–4.77)*^a^	0.98 (0.85–1.15)
*P* for trend	0.001	0.009	< 0.001	0.02	< 0.001	0.53
Anger In						
Low (referent)	1.0	1.0	1.0	1.0	1.0	1.0
Medium	1.07 (0.93–1.23)	1.04 (0.97–1.11)	1.01 (0.93–1.11)	0.97 (0.92–1.02)	1.60 (1.22–2.09)*^a^	0.98 (0.84–1.14)
High	1.37 (1.22–1.53)*^a^	1.05 (0.99–1.10)	1.06 (0.99–1.15)	0.89 (0.85–0.94)*^a^	1.81 (1.43–2.29)*^a^	0.88 (0.77–1.01)
*P* for trend	< 0.001	0.19	0.24	< 0.001	< 0.001	0.17
Anger Out						
Low (referent)	1.0	1.0	1.0	1.0	1.0	1.0
Medium	0.92 (0.80–1.05)	1.16 (1.09–1.23)*^a^	0.92 (0.84–1.01)	1.02 (0.97–1.07)	0.66 (0.49–0.88)*^a^	1.07 (0.94–1.23)^Ɨ^
High	1.23 (1.10–1.37)*^a^	1.01 (0.96–1.08)	1.04 (0.96–1.11)	0.96 (0.91–1.01)^Ɨ^	1.26 (1.03–1.53)*^a^	0.88 (0.77–1.02)
*P* for trend	< 0.001	< 0.001	0.03	0.05	< 0.001	0.04
Depressive symptoms						
Low (referent)	1.0	1.0	1.0	1.0	1.0	1.0
Medium	1.12 (0.99–1.26)	1.09 (1.02–1.16)*^a^	1.02 (0.95–1.10)	1.11 (1.05–1.17)*^a^	1.04 (0.81–1.32)	1.04 (0.89–1.20)
High	1.07 (0.94–1.21)	1.13 (1.06–1.21)*^a^	0.99 (0.92–1.08)	1.08 (1.02–1.13)*^a^	1.33 (1.06–1.67)*^a^	0.89 (0.76–1.03)
*P* for trend	0.15	0.001	0.76	< 0.001	0.02	0.07
Global Stress						
Low (referent)	1.0	1.0	1.0	1.0	1.0	1.0
Medium	1.21 (1.12–1.30)*^a^	1.05 (1.01–1.10)*	0.95 (0.91–0.99)	1.03 (1.00–1.06)^Ɨ^	0.97 (0.85–1.10)	1.19 (1.08–1.31)*^a^
High	1.26 (1.16–1.36)*^a^	1.16 (1.11–1.20)*^a^	1.10 (1.05–1.15)*^a^	1.02 (0.99–1.05)	1.01 (0.88–1.16)	1.27 (1.15–1.40)*^a^
*P* for trend	< 0.001	< 0.001	< 0.001	0.20	0.86	< 0.001
Weekly Stress-Event						
Low (referent)	1.0	1.0	1.0	1.0	1.0	1.0
Medium	1.19 (1.06–1.33)*^a^	1.08 (1.02–1.14)*^a^	0.88 (0.82–0.95)*^a^	0.97 (0.93–1.01)	1.08 (0.87–1.33)	0.70 (0.62–0.79)*^a^
High	1.04 (0.94–1.17)	1.11 (1.05–1.17)*^a^	0.96 (0.90–1.03)	0.96 (0.92–1.00)^Ɨ^	1.25 (1.03–1.52)*^a^	0.75 (0.67–0.85)*^a^
*P* for trend	< 0.001	< 0.001	0.003	0.10	0.08	< 0.001
Weekly Stress-Impact						
Low (referent)	1.0	1.0	1.0	1.0	1.0	1.0
Medium	1.09 (0.96–1.24)	0.96 (0.90–1.03)	1.00 (0.92–1.09)	0.95 (0.90–1.00)^Ɨ^	1.21 (0.91–1.60)	1.07 (0.91–1.27)
High	1.00 (0.86–1.15)	1.05 (0.98–1.12)	0.97 (0.88–1.06)	0.99 (0.94–1.04)	1.70 (1.31–2.21)*^a^	0.96 (0.80–1.14)
*P* for trend	0.33	0.04	0.72	0.15	< 0.001	0.41
**Major Life Events**						
Low (referent)	1.0	1.0	1.0	1.0	1.0	1.0
Medium	1.24 (1.14–1.34)*^a^	1.05 (1.00–1.09)	0.99 (0.94–1.05)	0.95 (0.91–0.98)*^a^	1.01 (0.86–1.18)	1.18 (1.07–1.30)*^a^
High	1.18 (1.09–1.28)*^a^	1.19 (1.14–1.23)*^a^	1.03 (0.98–1.09)	1.07 (1.04–1.10)*^a^	1.09 (0.94–1.26)	1.34 (1.22–1.48)*^a^
*P* for trend	< 0.001	< 0.001	0.37	< 0.001	0.53	< 0.001

*Bold indicates *p* value < 0.05. ^Ɨ^*P* value < 0.10. Abbreviations *CI* Confidence interval, *WSI* Weekly stress inventory, *SD* Standard deviation. Models adjusted for age, education, income, physical activity, smoking, fat intake, alcohol consumption and body mass index (hypertension and diabetes analysis). Multiple imputation for missing data in covariates based on 5 data sets. ^a^ Bonferroni Correction with *p* value < 0.02

Of the 5306 participants, 500 were excluded due to missing data for hypertension and diabetes (*n* = 214), education, smoking, physical activity and diet (*n* = 286), leaving 4806 (91% of the cohort) in the analytic sample. There were 16% missing values for income and between 9 and 40% missing on psychosocial variables. Therefore, we performed multiple imputations by chained equations (MICE) using five [5] data sets in order to reduce potential bias and loss of power. All reported *p* values correspond to 2-tailed tests and significant at the 0.05 level. Due to performing multiple test scenarios that put us at an increased risk for Type I error, we implemented Bonferroni method of correction and presented adjusted *p* values for multiple testing in the results. Analyses were performed using STATA 15.0 (StataCorp, College Station, TX).

## Results

**Fig. 1:**
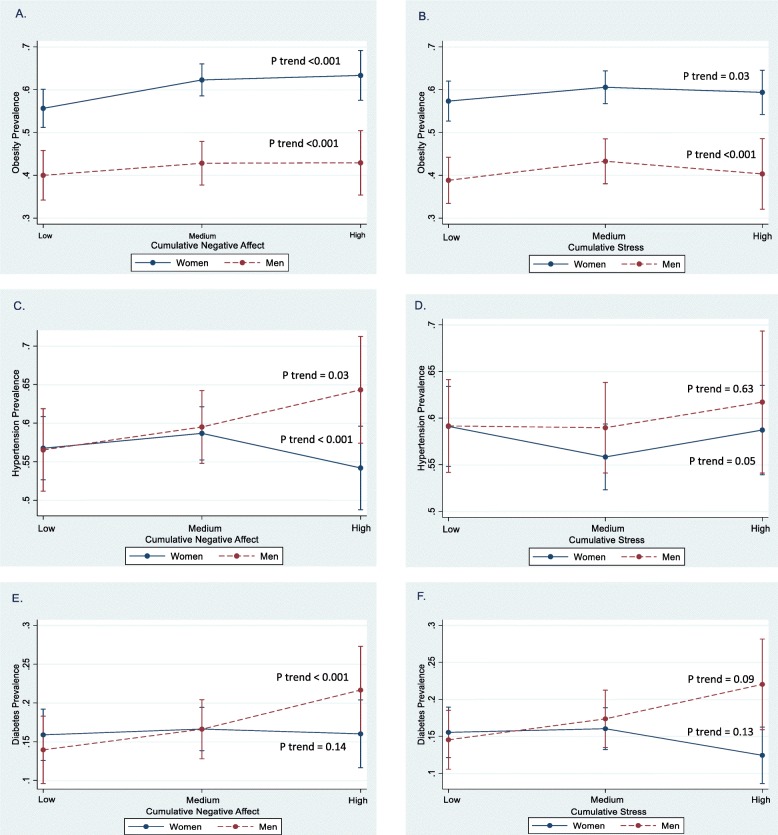
Predicted associations of obesity prevalence (**a** and **b**), hypertension prevalence (**c** and **d)** and diabetes prevalence (**e** and **f**) with levels of cumulative negative affect and cumulative stress by sex with 95% confidence intervals-CIs, JHS 2000–2004. *Notes:* Fully adjusted models of the association of predicted prevalence ratios (PR, 95% CI) of obesity, hypertension, and diabetes at baseline with low, medium, and high cumulative negative affect and cumulative stress by sex. All predicted models are adjusted for age, education, income, smoking, physical activity, fat in diet, alcohol consumption, and body mass index (except in the obesity analysis). *P* for trend represents the linear trend across categories of cumulative negative affect and cumulative stress stratified by women and men

Men reported higher cynicism than women (*p* < 0.001). Women reported greater depressive symptoms, global stress, WSI-impact, MLEs, and cumulative stress than men (*p* < 0.05). CVD risk factors were higher among women (*p* < 0.05) (Table 1). After full adjustment, men who reported high (vs. low) cumulative negative affect had a 36% increased prevalence of *obesity* (PR 1.36, 95% CI 1.16–1.60). Men and women who reported high (vs. low) cumulative stress had a 24 and 13% increased prevalence of obesity (PR 1.24, 95% CI 1.01–1.52; PR 1.13, 95% CI 1.03–1.23, respectively) (data not shown). Fig 1 a and b show that at higher levels of cumulative negative affect and cumulative stress, the prevalence of obesity is high, especially among women (p for trend < 0.05). Table 2 shows high cynicism, stress, and MLEs were positively associated with prevalent obesity in men and women (*p* < 0.05). Anger-in and -out were significant for men, while only depressive symptoms and WSI-events were significant for women (*p* < 0.05).

**Table 3:** Prevalence Ratios (PR 95% CI) of Hypertension and Diabetes Control by psychosocial measures among men and women, JHS (2000–2004).

*Psychosocial Measures*	Hypertension Control (PR, 95% CI)		Diabetes Control (PR, 95% CI)	
	Men	Women	Men	Women
Cynical distrust				
Low (referent)	1.0	1.0	1.0	1.0
Medium	0.75 (0.55–1.02)^Ɨ^	0.97 (0.77–1.23)	1.49 (0.70–3.22)	0.79 (0.65–0.95)*^a^
High	1.26 (0.95–1.68)	0.62 (0.46–0.84)*	1.70 (0.83–3.48)	0.49 (0.36–0.66)*^a^
*P* for trend	< 0.001	< 0.005	0.35	< 0.001
Anger In				
Low (referent)	1.0	1.0	1.0	1.0
Medium	1.04 (0.68–1.58)	1.27 (1.01–1.60)*^a^	1.85 (1.31–2.62)*^a^	1.48 (1.18–1.86)*^a^
High	1.44 (1.04–2.00)*	0.75 (0.60–0.95)	1.01 (0.70–1.47)	1.20 (0.92–1.55)
*P* for trend	0.05	< 0.001	< 0.001	0.003
Anger Out				
Low (referent)	1.0	1.0	1.0	1.0
Medium	0.72 (0.51–1.03)^Ɨ^	1.21 (0.99–1.47)*	2.63 (1.55–4.48)*^a^	0.57 (0.45–0.72)*^a^
High	1.28 (0.95–1.73)	0.77 (0.62–0.96)^Ɨ^	0.68 (0.50–0.93)*^a^	0.74 (0.59–0.92)*^a^
*P* for trend	0.01	< 0.001	< 0.001	< 0.001
Depressive symptoms				
Low (referent)	1.0	1.0	1.0	1.0
Medium	1.22 (0.90–1.66)	1.09 (0.85–1.38)	0.90 (0.64–1.27)	1.05 (0.83–1.33)
High	1.36 (1.00–1.85)^Ɨ^	1.24 (0.99–1.55)	0.76 (0.49–1.19)	0.94 (0.73–1.20)
*P* for trend	0.14	0.16	0.48	0.61
Global Stress				
Low (referent)	1.0	1.0	1.0	1.0
Medium	0.66 (0.55–0.78)*^a^	1.03 (0.91–1.18)	1.05 (0.81–1.36)	0.90 (0.78–1.03)
High	1.09 (0.92–1.29)	0.81 (0.71–0.94)*^a^	1.37 (1.05–1.78)	0.80 (0.68–0.95)*
*P* for trend	< 0.001	0.003	0.05	0.02
Weekly Stress-Events				
Low (referent)	1.0	1.0	1.0	1.0
Medium	0.84 (0.65–1.09)	0.93 (0.78–1.11)	1.32 (0.93–1.87)^Ɨ^	0.66 (0.53–0.82)*^a^
High	1.01 (0.78–1.30)	0.83 (0.68–1.01)	1.67 (1.09–2.57)*^a^	0.98 (0.82–1.17)
*P* for trend	0.35	0.19	0.06	< 0.001
Weekly Stress-Impact				
Low (referent)	1.0	1.0	1.0	1.0
Medium	1.68 (1.22–2.33)*^a^	0.86 (0.68–1.09)	0.82 (0.57–1.17)	0.65 (0.50–0.85)*^a^
High	1.95 (1.36–2.78)*^a^	0.97 (0.76–1.23)	0.93 (0.50–1.74)	0.72 (0.56–0.93)
*P* for trend	< 0.001	0.44	0.55	0.005
Major Life Events				
Low (referent)	1.0	1.0	1.0	1.0
Medium	0.94 (0.77–1.16)	0.87 (0.74–1.03)	0.90 (0.69–1.17)	1.02 (0.88–1.18)
High	1.30 (1.08–1.55)*	1.22 (1.08–1.39)*^a^	0.95 (0.70–1.28)	1.04 (0.87–1.24)
*P* for trend	0.08	< 0.001	0.74	0.90

*Bold indicates *p* value < 0.05. ^Ɨ^*P* value < 0.10. Abbreviations: *CI* confidence interval, *WSI* weekly stress inventory, *SD* standard deviation

Models adjusted for age, sex, education, income, physical activity, smoking, fat intake, alcohol consumption and body

mass index. Multiple imputation for missing data in covariates based on 5 data sets. ^a^ Bonferroni Correction with *p* value < 0.02

Among men, high cumulative negative affect was positively associated with *prevalent hypertension* (PR 1.16, 95% CI 1.04–1.30) (data not shown), also evident in Fig. 1 c that shows high cumulative negative affect results in hypertension prevalence just under 65%. There was a positive, dose-response gradient in levels of cynicism, and global stress with hypertension (*p* for trend < 0.05) (Table 2). Among women, high cumulative negative affect and cumulative stress (and anger-in) were associated with reduced hypertension prevalence (*p* < 0.05) (Fig. 1 c and d). High depressive symptoms and MLEs were positively associated with hypertension among women (Table 2). Predicted associations for men in Fig. 1 e and f show that at high levels of cumulative negative affect and high cumulative stress, *prevalent diabetes* was over 20%. For men, there was a stronger dose-response effect for each psychosocial measure except global stress and MLEs than for women (*p* for trend < 0.05) (Table 2). High stress and MLEs were positively associated with diabetes among women. Unexpectedly, high WSI-events were associated with a 25% reduced prevalence of diabetes for women (*p* < 0.05).

**Fig. 2:**
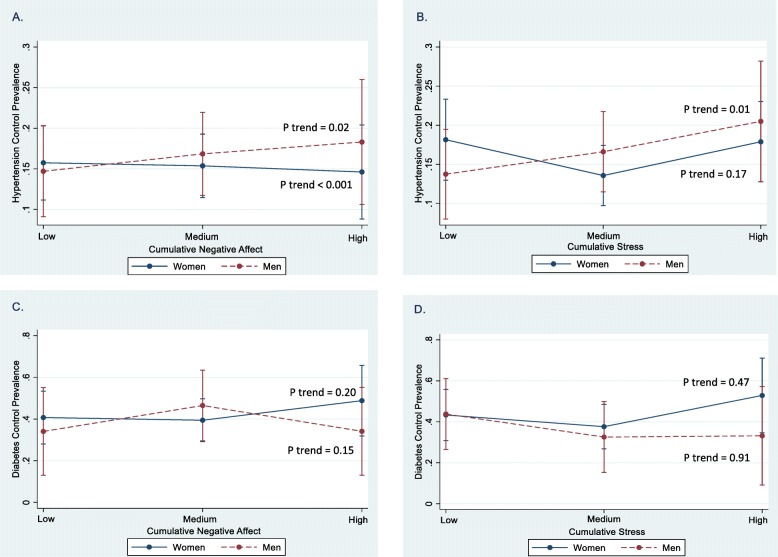
Predicted associations of prevalent hypertension control (**a** and **b**) and prevalent diabetes control (**c** and **d**) with levels of cumulative negative affect and cumulative stress by sex with 95% confidence intervals-CIs, JHS 2000–2004. *Notes:* Fully adjusted models of the association of predicted prevalence ratios (PR, 95% CI) of hypertension control and diabetes control at baseline with low, medium, and high cumulative negative affect and cumulative stress by sex. All predicted models are adjusted for age, education, income, smoking, physical activity, fat in diet, alcohol consumption, and body mass index. *P* for trend represents the linear trend across categories of cumulative negative affect and cumulative stress stratified by women and men

High (vs. low) cumulative negative affect and cumulative stress were positively associated with *hypertension control* among men (*p* < 0.05), while high cumulative negative affect was associated with reduced hypertension control among women (PR 0.40 95% CI 0.24–0.65) (data not shown). Fig. 2 a and b show that for men prevalent hypertension control increased at high levels of cumulative negative affect (18%) and cumulative stress (20%) (p for trend < 0.05). Men who reported high anger in, WSI-impact and MLEs had greater hypertension control, and women who reported high MLEs had greater hypertension control (Table 3). Cumulative negative affect or cumulative stress was not associated with *diabetes control* among men or women. Men who reported high anger-out had a 32% reduced prevalence of diabetes control, and high WSI-events were associated with diabetes control for men (Table 3). Conversely, cynicism, anger-out, and stress were associated with lower diabetes control among women (*p* < 0.05).

## Discussion

We analyzed associations of multiple psychosocial factors with CVD risk factors among African Americans. Cumulative negative affect and cumulative stress were more associated with risk factors for men than women. The majority of measures was associated with *obesity* among men and women. Individual negative affect and stress measures were equally associated with *hypertension* among men and women. Most negative affect and stress measures were positively associated with *diabetes* among men. Psychosocial measures were associated with lower *hypertension control* and *diabetes control* for women than men.

One study found that *obesity* was positively associated with depressive symptoms in a multi-ethnic sample of women (*n* = 1857) [22]. Another study of 112,716 men and women found that stress was associated with prevalent *obesity*, especially among women [23]. In our study, cumulative stress was equally associated with obesity among men and women; however, cumulative negative affect was only associated with obesity among men, which has not been studied in African Americans.

Few reports have examined multiple psychosocial factors and *hypertension* in large samples of African Americans. A JHS study found that high negative affect and stress measures were associated with risk of *BP progression* [13]. Spruill et al. [24] found that greater hostility and depressive symptoms were associated with reduced *BP dipping*. We found that high cynicism, anger-in and stress were associated with hypertension among men; and high depressive symptoms, stress, and MLEs were associated with hypertension among women. Studies examining psychosocial factors and *diabetes* have found that high anger temperament was associated with increased risk of diabetes among African Americans (aged 48–67; *n* = 11,615); and that high anger was associated with diabetes risk in a multi-ethnic sample (*n* = 5598 adults; 28% African Americans) [25, 26]. Similarly, we found an association with anger and higher diabetes among men. Research has also shown that stress was associated with prevalent diabetes among white adults aged 50–74 years [27]. We found that stressors were associated with diabetes among women, and cumulative stress was associated with diabetes among men.

Racial disparities in *hypertension control* are partly due to poor health behaviors and lower quality of care [28]; however, little research has examined whether hypertension control is associated with psychosocial factors. We found that women who reported high cynicism and cumulative negative affect had lower hypertension control, while men who reported high cumulative stress had greater hypertension control**.**

Regarding *diabetes control* one study found no association between work-related stress and HbA1c among blacks and whites (*n* = 537, aged 25–59) [29], while another study found that increased discrimination and stress were associated with poorer glycemic control among black and white diabetics (*n* = 615, 18 years and older) [30]. Our study used multiple psychosocial measures, while the previous smaller studies used only stress measures. Although women had greater diabetes control than men in the JHS (39% vs. 35%, respectively) [2], we found a greater number of psychosocial factors associated with lower diabetes control among women.

Two notable *pathways* may be considered in explaining our findings. First, *poor behaviors* may occur as a coping response to psychosocial exposure, such as increased smoking and alcohol consumption, decreased exercise and sleep, poorer dietary choices and non-adherence to medication regimens [31]. These factors in turn contribute to increased risk factors for heart disease. Poor behaviors likely impacted the sex differences in the association of cumulative negative affect and cumulative stress with obesity between men and women. For example, high stress may be related to physical inactivity and poor diet which are precursors to obesity. Unexpectedly, high cumulative negative affect and cumulative stress were associated with reduced prevalence of hypertension and diabetes, respectively, among women. Perhaps women, who report high negative affect and stress, cope and display psychosocial resources (e.g., social networks) differently compared to men which ultimately contribute to low hypertension and diabetes. Similarly, coping mechanisms may also lessen the impact stress has on the ability of men to manage hypertension. For example, men may manage stress differently with more physical activity [14], which may help to promote hypertension control.

Second, exposure to psychosocial stressors may cause emotional distress, which triggers *physiological responses* involving the hypothalamic-pituitary-adrenal (HPA) axis and the sympathetic-adrenal-medullary (SAM) system. The release of cortisol in the HPA activation leads to accumulation of visceral fat, cardiovascular reactivity, as evidenced by increased blood pressure and hyperglycemia [30]. Repeated activation of these systems can interfere with the normal control of physiological systems, and result in chronic illnesses.

The following limitations should be considered in the context of our findings. This study was conducted in a single metropolitan area, which limits its generalizability to other African American populations. Additionally, given the four recruitment strategies used in the JHS, selection bias may be a threat to internal validity. Moreover, all psychosocial measures were self-reported, and therefore potentially affected by reporting bias. The study design is cross-sectional which limited our ability to draw causal inferences. Bidirectional associations between psychosocial factors and outcomes were not tested and could be explored, particularly the obesity-depression/stress link, which has been established in the literature [32]. Strengths of this study include utilizing JHS, the largest study of CVD in African Americans. This study also examined multiple dimensions of negative affect and stress and CVD risk factors. We also examined cumulative stress and cumulative negative affect indices to capture how these factors act in clusters to uniquely impact CVD risk factors in African Americans. Finally, we examined the associations of psychosocial risk factors with *disease management* (hypertension control and diabetes control) in a large at-risk sample of African Americans. This is novel in that it shows psychosocial factors are key determinants of hypertension control and diabetes control.

## Conclusions

This study examined the relationship between psychosocial factors and CVD risk factors, as well as associations with *disease management*, which to our knowledge is the first to study psychosocial factors and management of CVD risk factors among African Americans. The joint accumulation of psychosocial factors was more associated with risk factors for men than women. Prevention intervention efforts may want to consider the joint accumulation of stress and negative affect African Americans experience in addressing disparities in risk factors and disease management. Another clinical implication is to consider the intersectionality of multiple psychosocial factors in treating CVD among African Americans.

## Data Availability

The dataset generated and/or analyzed during the current study are not publicly available due to the Data Materials and Distribution Agreement (DMDA) that governs data use for the JHS and precludes the author(s) from sharing data.

## Abbreviations

CVD: Cardiovascular disease
JHS: Jackson Heart Study
CHD: Coronary heart disease
MS: Mississippi
BMI: Body mass index
BP: Blood pressure
HbA1c: Hemoglobin A1c
WSI: Weekly stress inventory
MLEs: Major life events
CES-D: Centers for epidemiologic studies depression
ANOVA: Analysis of variance
PR: Prevalence ratio
CI: Confidence interval
MICE: Multiple imputations by chained equations
HPA: Hypothalamic-pituitary-adrenal
SAM: Sympathetic-adrenal-medullary
